# Fetal Alcohol Exposure Alters Proopiomelanocortin Gene Expression and Hypothalamic-Pituitary-Adrenal Axis Function via Increasing MeCP2 Expression in the Hypothalamus

**DOI:** 10.1371/journal.pone.0113228

**Published:** 2014-11-19

**Authors:** Omkaram Gangisetty, Rola Bekdash, George Maglakelidze, Dipak K. Sarkar

**Affiliations:** Endocrine Program, Department of Animal Sciences, Rutgers University, New Brunswick, New Jersey, United States of America; Radboud University, The Netherlands

## Abstract

Proopiomelanocortin (POMC) is a precursor gene of the neuropeptide β-endorphin in the hypothalamus and is known to regulate various physiological functions including stress response. Several recent reports showed that fetal alcohol exposure programs the hypothalamus to produce lower levels of POMC gene transcripts and to elevate the hypothalamic-pituitary-adrenal (HPA) axis response to stressful stimuli. We investigated the role of methyl CpG binding protein (MeCP2) in the effects of prenatal ethanol on POMC gene expression and hypothalamic-pituitary-adrenal (HPA) axis function. Pregnant Sprague Dawley rats were fed between GD 7 and 21 with a liquid diet containing 6.7% alcohol, pair-fed with isocaloric liquid diet, or fed *ad libitum* with rat chow, and their male offsprings were used at 60 days after birth in this study. Fetal alcohol exposure reduced the level of POMC mRNA, but increased the level of DNA methylation of this gene in the arcuate nucleus (ARC) of the hypothalamus where the POMC neuronal cell bodies are located. Fetal alcohol exposed rats showed a significant increase in MeCP2 protein levels in POMC cells, MeCP2 gene transcript levels as well as increased MeCP2 protein binding on the POMC promoter in the arcuate nucleus. Lentiviral delivery of MeCP2 shRNA into the third ventricle efficiently reduced MeCP2 expression and prevented the effect of prenatal ethanol on POMC gene expression in the arcuate nucleus. MeCP2-shRNA treatment also normalized the prenatal ethanol-induced increase in corticotropin releasing hormone (CRH) gene expression in the hypothalamus and elevated plasma adrenocorticotrophic hormone (ACTH) and corticosterone hormone responses to lipopolysaccharide (LPS) challenge. These results suggest that fetal alcohol programming of POMC gene may involve recruitment of MeCP2 on to the methylated promoter of the POMC gene to suppress POMC transcript levels and contribute to HPA axis dysregulation.

## Introduction

Alcohol exposure during pregnancy causes fetal alcohol spectrum disorder in the offspring characterized by various neural developmental deficits, growth retardation and facial abnormalities. A common endophenotype of fetal alcohol exposed offspring is an elevated neuroendocrine response of the HPA axis [1]–[3], particularly an increase in circulating ACTH and corticosterone, which has been suggested to be due, at least in part, to the deleterious effects of alcohol exposure on hypothalamic β-endorphin producing neurons [4], [5]. During the stress response, hypothalamic peptides are released through several signaling cascades, such as the release of corticotropin-releasing hormone (CRH) followed by the release of various POMC-derived peptides. POMC is a relatively large peptide that is cleaved into multiple biologically active subunits, including β-endorphin and α-melanocyte stimulating hormone (α-MSH). Upon stimulation, β-endorphin synthesis, primarily within the arcuate nucleus of the hypothalamus, is activated by CRH release from terminals emerging from the paraventricular nucleus (PVN) of the hypothalamus, which is in turn inhibited by β-endorphin release [3], [6]. POMC-derived neuropeptides play a vital role in many other processes such as energy homeostasis, stress response, immune functions and the brain reward system [5], [7]–[9]. POMC system abnormalities have been associated with stress dysregulation, metabolic diseases, cancer and alcohol drinking [10]–[15].

The molecular regulatory mechanism controlling POMC expression with fetal alcohol exposure is not clearly understood. Aberrant epigenetic changes in response to environmental exposure in the uterus during fetal development are considered a potential mechanism [16], [17]. Epigenetic alterations including DNA methylation, histone code modifications and micro RNAs play an important role in regulating gene expression. Epigenetic regulation of POMC gene expression in obesity associated metabolic syndrome, lung cancer and Cushing's syndromes have been reported [18]–[20]. We recently showed increased promoter DNA methylation in the POMC is due to altered DNA methyl transferase (DNMT) and histone deacetylase (HDAC) activity in POMC-producing cells in the arcuate nucleus of the hypothalamus in fetal alcohol exposed rats [17], [21]. It has been reported that methylated promoters recruit methyl CpG binding domain (MBD) containing proteins to silence gene expression [22]. MeCP2 is a founding member in this family of proteins, possessing a MBD motif which can recognize and bind methylated DNA thus recruiting additional transcriptional repressors such as HDACs to maintain repressive chromatin function [23]. In this study we tested the hypothesis that fetal alcohol exposure recruits increased amounts of MeCP2 on to the methylated promoter of the POMC gene to suppress POMC transcript levels and increase the HPA axis response to a stress challenge.

## Materials and Methods

### Ethics statement

Animal surgery and care were performed in accordance with institutional guidelines and complied with the National Institutes of Health (NIH) policy. All experimental procedures and animal treatment protocols were approved by Rutgers Animal Care and Facilities Committee and complied with NIH policies.

### Animal and alcohol feeding

Sprague-Dawley rats were obtained from Charles River (Wilmington, MA), housed in controlled conditions with a 12 h light/dark cycle at a constant temperature 22°C throughout the study. These rats were bred in our animal facility. On gestational day 7 through 21, pregnant rats were fed rat chow ad libitum (AD), a liquid diet containing ethanol (AF; Bioserve, Frenchtown, NJ) or pair-fed (PF) an isocaloric liquid control diet (with ethanol calories replaced by maltose-dextrin; Bioserve). To habituate the dams with an alcohol diet, they were fed a liquid diet containing 1.7% (v/v) ethanol on day 7 and 8 and then 5.0% (v/v) ethanol on day 9 and 10. From days 11 to 21, animals were fed with 6.7% ethanol (v/v) providing about 35% of the total dietary calories. Previous studies have shown that the peak blood ethanol concentration is achieved in the range of 120–150 mg/dl in pregnant dams fed with this liquid diet [24]. After birth, AF and PF pups were cross fostered with the rat chow fed (AD) fostering dams until postnatal day 22 to control the possible effect of changes in maternal behavior or physiology due to alcohol withdrawal after birth. Pups were then weaned and subsequently housed by treatment and sex and provided with rat chow and water *ad libitum* for the duration of the study. Male offspring from each litter were selected for each experimental group in order to prevent any gene homogeneity. After 60 days of birth, rats were euthanized and brain tissues were collected and stored at −80°C until use.

### Lentivirus transduction and delivery into the rat third ventricle

Lentiviral vector plasmids carrying scrambled short hairpin RNA (scr-sh), MeCP2 shRNA and supporting plasmids (Vsvg, del 8.9) were received as a kind gift from Dr. Anne E. West from Duke University, Durham, NC [25]. The sequences for scr-sh and MeCP2 sh RNA are listed in the Table 1. Plasmid DNA was transformed into Stable3 competent bacterial cells as per the instructions from the manufacturer (Invitrogen, Grand Island, NY). Plamid DNA was extracted using a midi plasmid DNA extraction kit (Qiagen Valenica CA). Lentiviral plasmid DNA along with supporting plasmids were tranfected into HEK293 FT cells using lipofectamin LTX reagent following the instructions from the manufacturer (Invitrogen, Grand Island, NY). Lentiviral supernatant media samples produced from transfected cells were collected 48 h after transfection and centrifuged at 2000×g for 7 min and filtered through 0.4 µm filter. Lentivirus was concentrated by high-speed centrifugation at 25000 rpm at 4°C for 90 min in an ultra-centrifuge (Beckman, Brea, CA) in sterile PBS. The titer for concentrated lentivirus produced was determined by infecting the serially diluted virus along with transfection reagent polybrene to HEK293FT cells. The infected cells were observed under fluorescent microscopy for green fluorescence, since these viral vectors co-expresses green fluorescent protein (GFP) in their constructs. The titer was found to be 2×10^6^ pfu/µl. *In vitro* validation of the MeCP2 sh-RNA lentivirus was performed by infecting the virus to primary pituitary PR1 cells. In vivo validation was performed by delivering the virus into the rat third ventricle and localizing the GFP labeled cells in the ARC.

**Table 1:** Primer sequences.

Primer Name	Sequence
POMC FP	5′-CAAGAGGGAGCTGGAAGGCGAGC-3′
POMC RP	5′-TCACTGGCCCTTCTTGTG-3′
MeCP2FP	5′-CAAACAGCGACGTTCCATCA-3′
MeCP2 RP	5′-TGTTTAAGCTTTCGCGTCCAA-3′
GAPDH FP	5′-AGACAGCCGCATCTTCTTGT-3′
GAPDH RP	5′-CTTGCCGTGGGTAGAGTCAT-3′
POMC MFP	5′-GTTAGGTGTGCGTTTTAGC-3′
POMC MRP	5′-CTAACAACGCTTCTACAACG-3′
POMC UFP	5′-GGGTTAGGTGTGTGTTTTAGT-3′
POMC URP	5′-CCTAACAACACTTCTACAACACA-3′
Scr-sh	5′-AAACAAGCCCATTCGCGGATT-3′
MeCP2-sh	5′-GTCAGAAGACCAGGATCTC-3′
POMC PFP	5′-GATTCGCTTGTTGCGTTG-3′
POMC PRP	5′- GCCTCTCTTAGTCACTGCTCC-3′
Control PFP	5′- GAGGTGCGGGCCATCAGCAG-3′
Control PRP	5′- AGTGCAGGGGGCTGGTCTCA-3′

Forward primer (FP), Reverse primer (RP), Methyl forward primer (MFP), Methyl reverse primer (MRP), Un methyl forward primer (UFP), Un methyl reverse primer (URP), Promoter forward primer (PFP), Promoter reverse primer (PRP).

The lentivirus was surgically injected into the third ventricle of fetal alcohol exposed (AF) and control-fed rats (AD, PF) 60 days after birth. Rats were anaesthetized with sodium pentobarbital and placed on a stereotaxic frame. A small incision was made on the skin that overlies the skull. A small hole was drilled in the skull using stereotaxic coordinates (1.8 mm posterior from the bregma and 8 mm below the cortex). A Hamilton syringe carrying either scr-sh (control) or MeCP2-sh lentivirus was lowered in the hole through which 5 µl virus (10×10^6^ pfu) was injected at the rate of 1 µl/min. After injection the syringe was kept in place for 5 min to avoid virus being sucked out during removal. The skin was closed with a wound clip. Rats were given two weeks time to recover and then used to collect the samples for gene transcript measurements by quantitative RT-PCR and gene methylation analysis by methylation specific PCR (MS-PCR).

A separate group of the AF, PF and AD rats treated with the MeCP2-sh or scr-sh (control) lentivirus were used in an immune stress experiment. Animals were injected intraperitoneally with 100 µg/kg body weight of lipopolysaccharide (LPS; Sigma Aldrich, St. Louis, MO) in saline or with saline alone as a control. After 2 hrs, trunk blood samples were collected in tubes containing ethylenediaminetetracetic acid (EDTA) and centrifuged at 2,000 rpm for 15 minutes to prepare plasma samples, which were used to determine hormone assays. Plasma samples were used for determination of corticosterone or ACTH levels using the rat corticosterone enzyme-linked immunoassay (IBLAmerica, Mineopolis, MN) and rodent ACTH ELISA (Phoenix Pharmaceuticals; Burlingame, CA) following the manufacturer's instructions.

### Reverse transcription polymerase chain reaction (RT-PCR)

Gene expression levels of POMC, MeCP2 and CRH in the hypothalamic tissues were measured by quantitative real time PCR (SYBR green assay). Total RNA of each mediobasal hypothalamus (MBH) sample was extracted using RNeasy purification kit (Quiagen, Valenica CA) and converted to first strand complementary DNA (cDNA) using high capacity cDNA reverse transcription kit (Applied Biosystems, Carlsbad, CA). The primer sequences used for POMC, MeCP2, CRH and the housekeeping gene GAPDH are as described previously [21] and given in Table 1. RT- PCR was performed at 95°C for 5 min followed by 40 cycles of 95°C for 15 sec, 60°C for 30 sec, 72°C for 40 sec in Applied Biosystems 7500 Real time PCR system (ABI Carlsbad, CA). The quantity of target gene expression was measured using a standard curve. The relative expression level of each hypothalamic gene was quantitated as the ratio of GAPDH.

### Western blot analysis

Protein levels of MeCP2 were determined by western blot analysis. MBH tissue samples were processed for protein extraction followed by quantification of total protein levels by Bradford Assay (Bio-Rad Laboratories, Hercules, CA). About 30 µg of total protein was run in 12% SDS PAGE and transferred to PVDF membrane at 30 V overnight at 4°C. The membranes were blocked in 5% nonfat dry milk-TBS-0.1% Tween 20 (TBST) at room temp for 2 h. The membranes were incubated with primary antibody in the same blocking buffer at 4°C overnight. The primary antibodies used were rabbit anti-MeCP2 (1∶1000; EMD Millipore, Billerica, MA), and mouse anti-actin antibody (1∶5000; Vector labs, Burlingame, CA). The membranes were washed in TBST and then incubated with corresponding peroxidase conjugated secondary antibody at room temperature for 1 h. The membranes were washed in TBST and incubated with ECL reagent and were developed on the film using X-ray developer. The protein band intensities were determined by Image J analysis software (National Institutes of Health, Bethesda, MD). Protein band intensity was normalized with corresponding actin band intensity.

### Methylation specific (MSP) RT-PCR

POMC methylation was performed by SYBR green real time PCR method of methylation specific PCR (MSP) as it was described previously [21]. DNA was extracted from MBH samples using DNeasy purification kit (Quiagen, Valenica CA). The bisulfite conversion of DNA was performed using EZ DNA methylation kit (Zymo Research, Orange, CA). Primer sequences specific for methylated and unmethylated DNA were used as described previously [21] and listed in Table 1. Rat highly methylated, low methylated control DNA (Epigen Dx, Worcester MA) also underwent bisulfite conversion and were used for preparing a standard curve. Real time quantitative PCR was performed at 95°C for 5 min followed by 40 cycles of 95°C for 15 sec, 60°C for 30 sec, 72°C for 40 sec. Relative quantity of methylated and unmethylated DNA were measured using a standard curve. DNA methylation was measured as a ratio of methylated versus unmethylated DNA.

### Double-immunostaining for MeCP2 and β-endorphin

Immunostaining for MeCP2 and β-endorphin were performed as described previously [17]. Brain sections (20 µm thickness) were cut on a cryostat (Leica) and collected on pre-chilled glass slides. These sections were double stained for MeCP2 (1∶500) and β-endorphin (1∶200) antibodies. The primary antibodies used were mouse anti MeCP2 (Abcam, Cambridge, MA) and rabbit anti β-endorphin (Bachem, Sam Carlos, CA). Secondary antibodies used in this study were Alexa-Flour 488 donkey anti-mouse (2 mg/ml; Invitrogen, Grand Island, NY) and Alexa Flour 594 donkey anti-rabbit IgG (2 mg/ml; Invitrogen, Grand Island, NY). After staining, pictures were taken using a confocal microscope with a 20× objective (Nikon EZ-C1 3.60 build 770, Gold version; Melville, NY). Total number of β-endorphin cells as well as total number of β-endorphin cells, located on the right and left side of the third ventricle, that were positive for MeCP2 were counted. The experimenters were blind to the experimental treatment group of the section during counting.

### Chromatin immunoprecipitation (ChIP) assay

ChIP assays were performed using a ChIP Assay Kit (EMD Millipore, Billerica, MA) following the instructions from the manufacturer. Micropunches of PVN and ARC were prepared from the hypothalamus starting from Bregma −0.8–−2.12 and −2.12–−4.52 mm, respectively. The punches from four animals were pooled to run a single immunoprecipitation. Proteins were cross-linked in DMEM plus 1% formaldehyde at 37°C. Protease inhibitor cocktail (Roche Applied Science, Indianapolis, IN,) was added to the lysis buffer and ChIP dilution buffer. Crosslinked DNA was sheared to approximately 300–700 base pairs with five sets of 5-s pulses using a Misonix Sonicator 4000 (Fisher scientific, Pittsburgh, PA), 5 mm tip and set to 50% maximum power. Sonicated cell supernatant was diluted 10-fold in ChIP dilution buffer. A sample was removed after dilution and called the “input” sample. The remaining diluted cell supernatant sample was pre-cleared with Salmon Sperm DNA/Protein A agarose 50% slurry. Primary MeCP2 antibody (1∶1000) (EMD Millipore, Billerica, MA) was added to the remaining precleared supernatant and incubated overnight. Equal concentration of the normal Rabbit IgG (Cell Signaling Technology, Danvers, MA,) was employed as a negative control. Rabbit IgG agarose beads were added and incubated by mixing on a rotator at 4°C. Agarose beads were washed, DNA was eluted after reverse cross linking with proteins. Real-time PCR was performed with the resulting sample as well as on the negative control. Serial dilutions of the “input” samples were used as a standard curve. PCR conditions were: 95°C for 2 min, 30 cycles of 95°C 30 s, 60°C for 30 s, 72°C for 30 s, subsequently followed by 72°C for 10 min. The results were normalized relative to the control gene. The control gene sequence is ∼1 kb downstream of the GAPDH transcription start and does not bind MeCP2 in rodents [23]. The primer sequences for the POMC promoter and GAPDH controls were listed in Table 1.

### Statistical analysis

The data shown in the figures are mean ± SEM. The significant differences between different treatment groups were assessed with one-way analysis of variance (ANOVA) with post-hoc analysis using the Newman Keuls post-test. Two-way ANOVA with Bonferroni post-hoc test was used to assess the differences between the groups of ACTH and corticosterone values. P<0.05 was considered significant.

## Results

### Fetal alcohol exposure alters POMC expression and methylation in the hypothalamus

**Figure 1:**
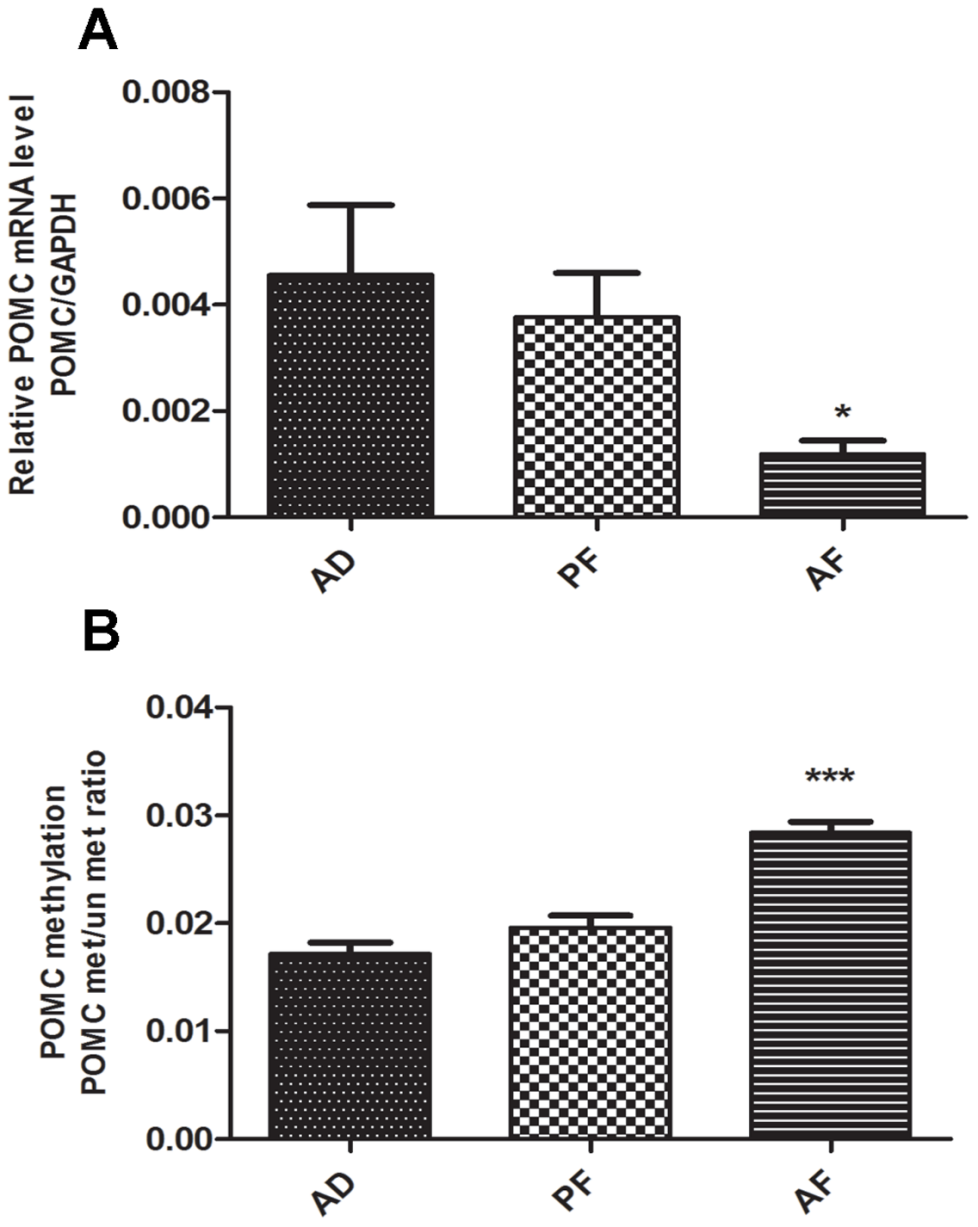
POMC gene expression and methylation changes in MBH of fetal alcohol exposed rat offspring. **A**. POMC mRNA levels in MBH of AD, PF and AF rat offspring were measured by quantitative RT-PCR and the amounts were normalized with GAPDH values and expressed as relative mRNA levels. Data are mean ± SEM (n = 6) and were analyzed using one-way ANOVA with Newman-Keuls post-hoc test; *, *P*<0.05, AF vs AD or PF. **B**. POMC promoter methylation levels in MBH of AD, PF and AF rat offspring were assayed by real time methylation specific PCR. POMC promoter methylation levels were measured as ratios of methylated verses unmethylated DNA values. Data are mean ± SEM (n = 6) and were analyzed using one-way ANOVA with Newman-Keuls post-hoc test; ***, *P*<0.001, AF vs AD or PF.

We reported previously that alcohol feeding using the liquid diet did not change body weight or litter size of dams and body weight of offspring [21]. Using this animal model of alcohol feeding, we show here that fetal alcohol exposed offspring had reduced levels of POMC gene expression (Fig. 1A) and increased levels of POMC promoter methylation in MBH tissue (Fig. 1B). Our previous results also showed the same trend in arcuate nucleus [21]. These results are consistent with previous reports that fetal alcohol exposure epigenetically programs the POMC gene to express its transcript in a lower level [17], [21].

### Fetal alcohol exposure increases MeCP2 expression and its binding to the POMC promoter in the hypothalamus

**Figure 2:**
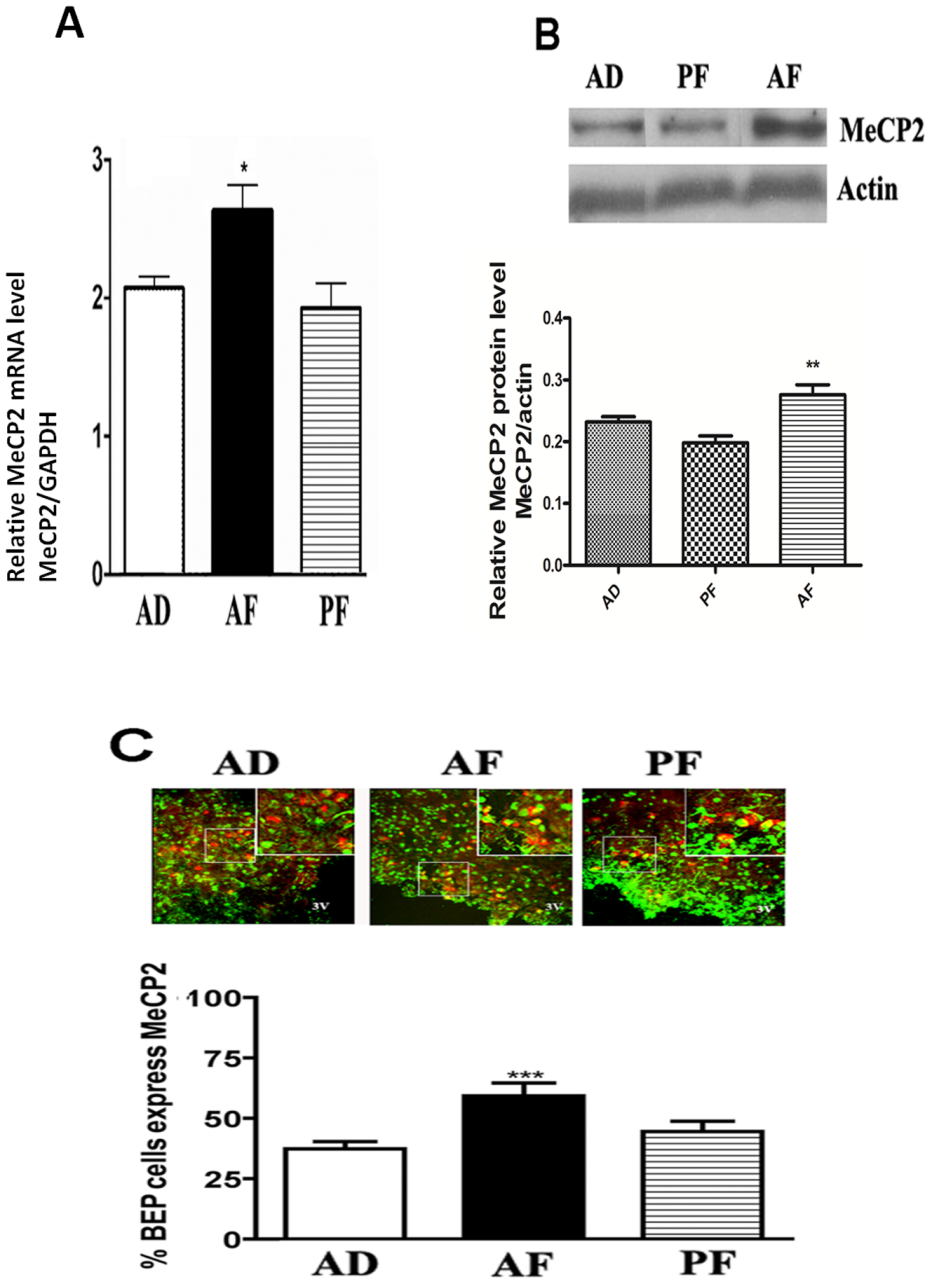
Changes in MeCP2 gene and protein levels in MBH of fetal alcohol exposed rat offspring. **A**. MeCP2 mRNA levels in MBH of AD, PF and AF rat offspring. MeCP2 mRNA levels were measured by quantitative RT-PCR and the amounts were normalized with GAPDH values and expressed as relative mRNA levels. Data are mean ± SEM (n = 6) and were analyzed using one-way ANOVA with Newman-Keuls post-hoc test; *, *P*<0.05, AF vs. AD or PF. **B**. A representative western blot gel demonstrating changes in MeCP2 protein levels (top) and actin levels (loading control, bottom) in MBH of AD, PF and AF rat offspring along with quantification measurements were represented as relative protein level (MeCP2/actin). Data are mean ± SEM (n = 6) and were analyzed using one-way ANOVA with Newman-Keuls post-hoc test; **, *P*<0.01, AF vs. AD or PF. **C**. MeCP2 protein levels in β-endorphin neurons in the ARC of AD, PF and AF rat offspring were measured by double immunofluorescence methods (MeCP2 proteins are shown in green and β-endorphin shown in red). Representative photographs show double-labeled cells in each group. Histograms show the mean ± SEM (n = 6) values of percent β-EP cells expressing MeCP2 and were analyzed using one-way ANOVA with Newman-Keuls post-hoc test; ***, *P*<0.001, AF vs. AD or PF.

We next sought to determine the role of MeCP2 in fetal alcohol exposure-induced repression of POMC expression. Methyl CpG binding domain (MBD) proteins are known to bind hypermethylated promoters thereby repressing transcription. MeCP2 has a conserved MBD motif which specifically recognizes and binds methylated DNA [26]. In our studies we found that MeCP2 mRNA expression and protein were significantly increased in the MBH of AF rats compared to AD and PF control rats (Fig. 2A,B). The protein level of MeCP2 was also increased in β-endorphin cells of the ARC of AF rats, as compared to AD and PF rats (Fig. 2C).

**Figure 3:**
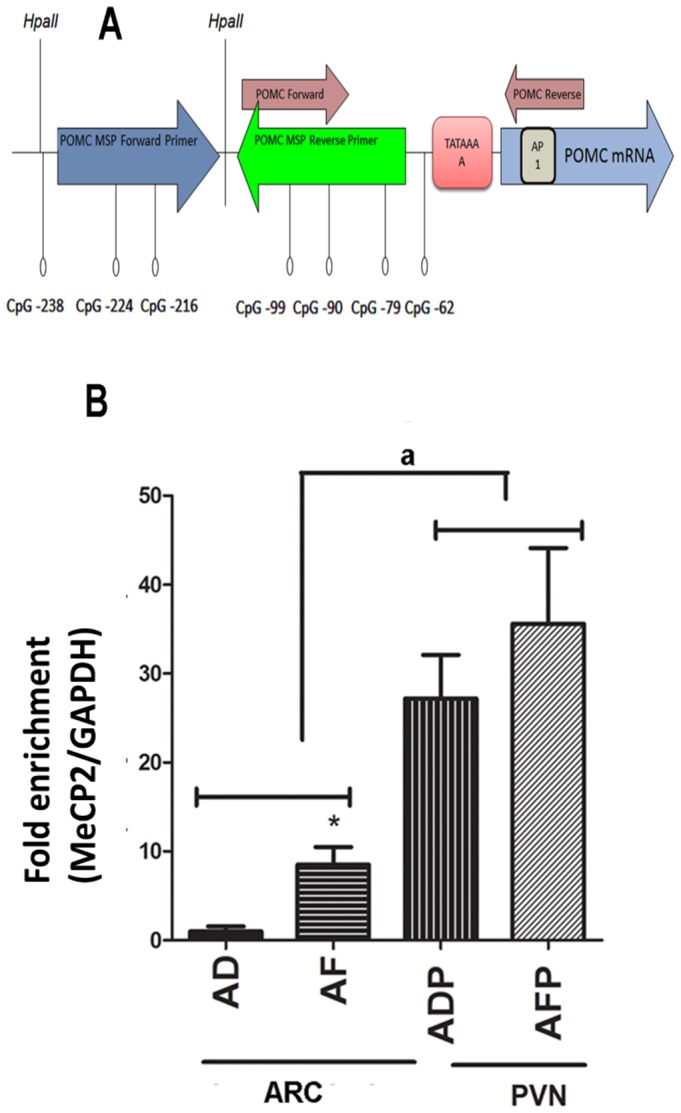
Changes in MeCP2 binding onto POMC promoter in ARC and PVN of fetal alcohol exposed rat offspring. **A**. Schematic representation of POMC promoter with CpG sites and primers flanking MeCP2 binding site. **B**. The levels of MeCP2 binding on POMC promoter in ARC and PVN of AD, PF and AF rat offspring were measured by ChIP assay. MeCP2 bound DNA pulled by its specific antibody was amplified by real time PCR using primers specific for the POMC promoter. MeCP2 enriched DNA was normalized with GAPDH and expressed as fold enrichment (MeCP2/GAPDH). AD and AF are ARC samples of *ad libitum*-fed and alcohol-fed rats. ADP and AFP are PVN samples of *ad libitum*-fed and alcohol-fed rats. Data are mean ± SEM (n = 6) and were analyzed using one-way ANOVA with Newman-Keuls post-hoc test; *, *P*<0.05, AF vs. AD; ^a^, P<0.001, ADP and AFP versus AD or AF.

Methylated CpG dinucleotides with adjacent A/T-rich sequences are often recognized by MeCP2 [27]. The proximal POMC promoter contains a number of A/T-rich stretches in the vicinity of CpG sites. We used the the ChIP assay technique to analyze the interaction between these regions and MeCP2. The primer binding sites covering CpG dinucleotides of the POMC promoter is represented in Fig. 3A. MeCP2 enrichment on the POMC promoter was increased significantly in the ARC of AF rats compared to AD rats (Fig. 3B). The PVN area where the POMC gene is not expressed was used as control tissue. MeCP2 showed strong binding on the POMC promoter in the PVN tissue. MeCP2 enrichment was unaltered in the PVN of AF rats (Fig. 3B). These results suggest that fetal alcohol exposure-induced increases in MeCP2 expression and its binding on the POMC promoter may play a role in repressing POMC expression.

### MeCP2 knock down normalizes POMC expression in the hypothalamus of fetal alcohol exposed animals

**Figure 4:**
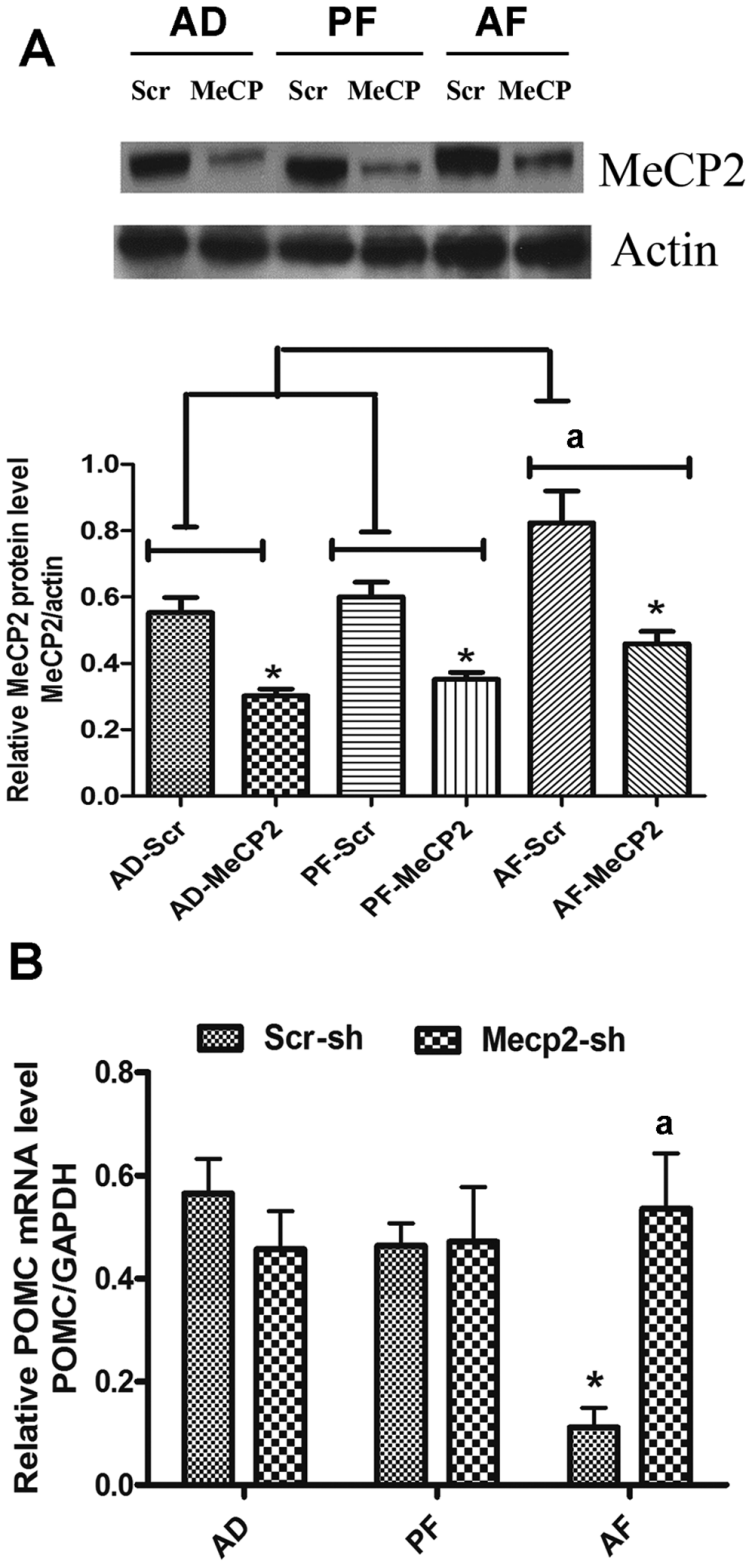
Effects of lentiviral knockdown of MeCP2 on POMC gene expression levels in fetal alcohol exposed rat offspring. **A**. A representative western blot gel showing MeCP2 protein levels (top) and actin levels (bottom) in MBH of scr sh (scr) or MeCP2 sh (MeCP) RNA-treated AD, PF and AF rat offspring. Histograms showing MeCP2 and actin ratio values in MBH of AD, PF and AF rats.treated with scr sh or MeCP2 sh RNA. Data are mean ± SEM (n = 6) and were analyzed using one-way ANOVA with Newman-Keuls post-hoc test; *, P<0.05, MeCP2 sh vs. scr-sh; ^a^, P<0.05, AF vs. AD or PF. **B**. POMC mRNA levels in MBH of scr-sh and MeCP2 sh RNA of AD, PF and AF rat offspring. POMC mRNA amounts were normalized with GAPDH and expressed as relative mRNA level. Data are mean ± SEM (n = 6) and were analyzed using one-way ANOVA with Newman-Keuls post-hoc test; *, P<0.05, AF- vs. AD- or PF-scr-sh-treated groups; ^a^, P<0.05, MeCP2 sh vs. scr-sh (AF group).

Because MeCP2 expression and its binding was increased with prenatal ethanol exposure, we hypothesized that MeCP2 is involved in regulating POMC expression. To test this hypothesis we knocked down MeCP2 expression using shRNA specific to MeCP2 by delivering a lentivirus (10 million pfu) into the third ventricle of AF, AD and PF rat offspring. The results show that MeCP2 expression in the MBH was significantly reduced following MeCP2 shRNA treatment compared to scrshRNA treatment in AD, PF and AF rat offspring (Fig. 4A). Determination of POMC expression in MBH samples of scr shRNA and MeCP2 shRNA treated rats revealed that MeCP2 shRNA treatment increased POMC mRNA expression in AF but not in AD and PF rats (Fig. 4B). Additionally, POMC mRNA levels in MeCP2 shRNA-treated AF rats were comparable to those of scr shRNA and MeCP2 shRNA treated AD and PF rats. These results suggest that MeCP2 plays an important role in fetal alcohol-induced suppression of POMC gene.

### MeCP2 knock down normalizes stress axis hyperresponse in fetal alcohol exposed animals

**Figure 5:**
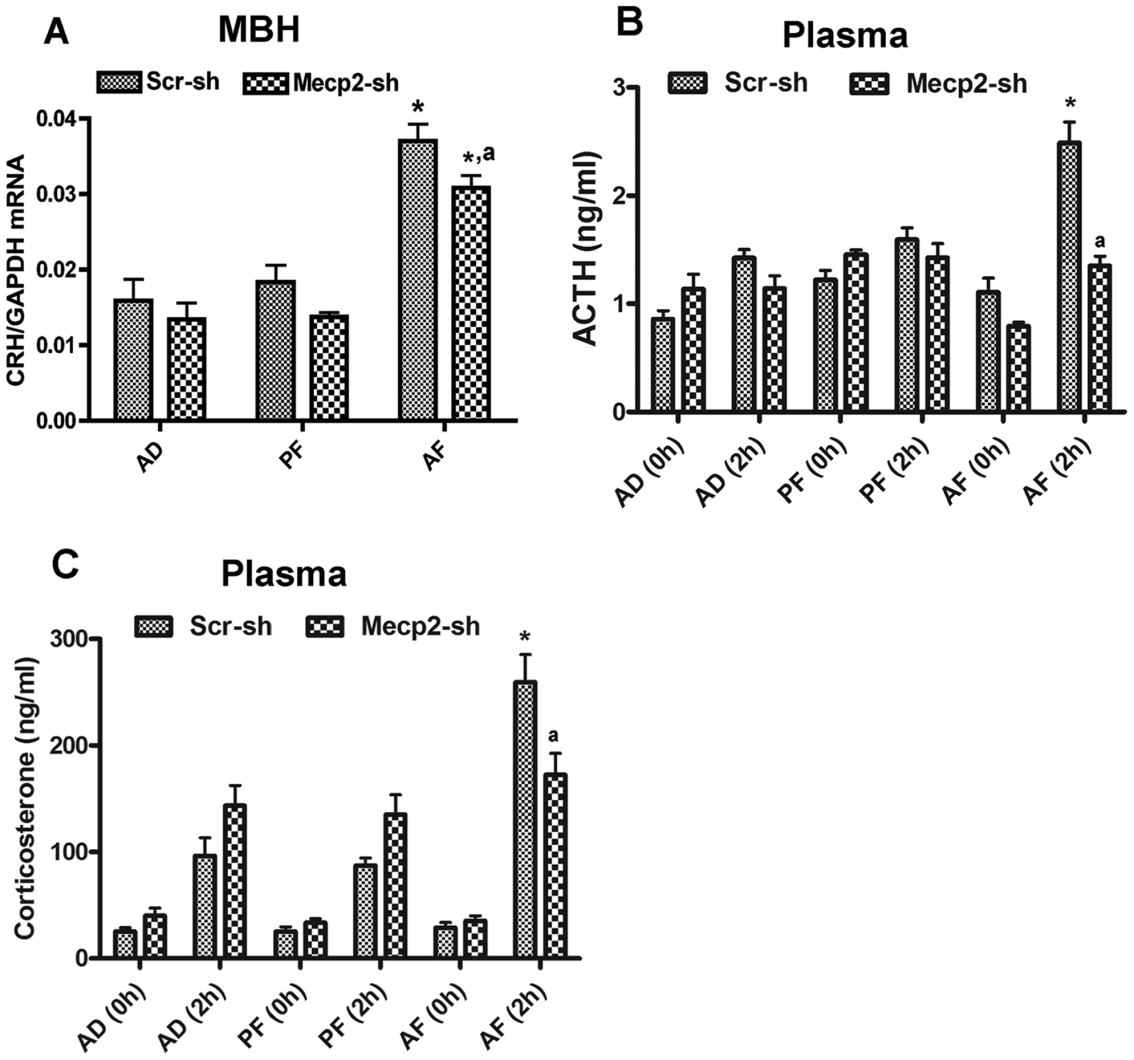
Effect of MeCP2 knockdown on stress axis responsiveness in fetal alcohol exposed rat offspring. **A**. CRH mRNA levels in the MBH of scr sh- or MeCP2 sh RNA-treated AD, PF and AF rat offspring. CRH mRNA amounts were normalized with GAPDH and expressed as relative mRNA level. Data are mean ± SEM (n = 6) and were analyzed using two-way ANOVA with Newman-Keuls post-hoc test; *, P<0.05, AF vs. AD or PF groups; ^a^, P<0.05, MeCP2 sh vs. scr-sh (AF group). **B**. ACTH levels in plasma of AD, PF or AF rats treated with either scr sh or MeCP2-sh RNA at 0 h and 2 h after LPS treatment. Data are mean ± SEM (n = 6) and were analyzed using two-way ANOVA with Bonferroni post-hoc test; *, P<0.05, AF vs. AD or PF (within Scr sh treatment groups); ^a^, P<0.05, MeCP2 sh vs. scr-sh (AF groups). **C**. Corticosterone levels in AD, PF or AF rats treated with either scr sh or MeCP2 sh RNA at 0 h and 2 h after LPS treatment. Data are mean ± SEM (n = 6) and were analyzed using two-way ANOVA with Bonferroni post-hoc test; *, P<0.05, AF vs. AD or PF (within Scr sh treatment groups); ^a^, P<0.05, MeCP2 sh vs. scr-sh (AF groups).

An endophenotype of the fetal alcohol effect on POMC gene is the hyperresponse of the HPA axis to stress [4], [5], [8]. We hypothesized that the MeCP2 normalizing effect on POMC gene expression would be reflected in the HPA axis response to an immune challenge. Previously, we have shown that fetal alcohol exposure increases CRH levels in the MBH and increases plasma ACTH and corticosterone responses to an LPS challenge [21]. In agreement with our previous study, we show here that fetal alcohol exposure increased CRH levels in the MBH (Fig. 5A) and increased plasma levels of ACTH and corticosterone 2 h after an LPS injection relative to control scr shRNA treated rats (Fig. 5B, C). In addition, we show here MeCP2 shRNA treatment prevented fetal alcohol effect on hypothalamic CRH and plasma ACTH and corticosterone response to LPS challenge. These results suggest that MeCP2 plays an important role in fetal alcohol-induced suppression of POMC gene expression and its control of the HPA axis function.

## Discussion

Fetal alcohol exposure is known to alter HPA axis activity partly via the suppression of POMC neuronal functions during adulthood [4], [28], [29]. We showed here that alcohol induced alteration of POMC neuronal functions involve hypermethylation of the POMC gene promoter and suppression of POMC gene transcription. We also provide evidence for a role of MeCP2 by showing an increased level of MeCP2 expression in POMC neurons in the hypothalamus of AF rats and by demonstrating a normalizing effect of MeCP2 shRNA on fetal alcohol-induced suppression of POMC gene expression and the HPA axis hyperresponse to LPS. These results suggest that MeCP2, a member of the MBD containing family of proteins, plays an important regulatory role in the epigenetic mechanism involved in fetal alcohol-induced alteration in POMC gene expression and its control of HPA axis function.

MeCP2 is abundantly expressed in the mature brain specifically in the hypothalamus and can either repress or activate gene expression [30], [31]. It has also been implicated in the behavioral response of rodents to drugs of abuse. For example, Im et al., 2010 [32] showed that knockdown of MeCP2 in the dorsal striatum decreased rat's intake of cocaine and implicated MeCP2 in the compulsive response of rats to this drug. Furthermore, they showed that cocaine stimulation of MeCP2 decreased the expression of miR-212 and miR-132 resulting in an increase of BDNF expression with an increase in cocaine intake. Moreover, MeCP2 has been implicated in stress regulation. Fyffe et al., 2008 [33] showed that MeCP2 knockout in the hypothalamus of mice resulted in specific behavioral phenotypes such as increased aggression, anxiety, abnormal response to stress and hyperphagia which are also endophenotypes observed in fetal-alcohol exposed rodents and human subjects [2], [34]. MeCP2 regulates chromatin structure of several genes and modulates their expression [30], [32]. We have previously shown that fetal alcohol exposure increases DNA methyltransferases (Dnmt)1 and the histone repressive mark H3K9me2 levels in POMC producing cells in the hypothalamus [17]. In this study, we showed fetal alcohol treatment increases MeCP2 protein levels in POMC cells as well as increases MeCP2 binding to the POMC gene promoter. These results could suggest that MeCP2 might play a role in POMC gene suppression in the hypothalamus by fetal alcohol exposure.

**Figure 6:**
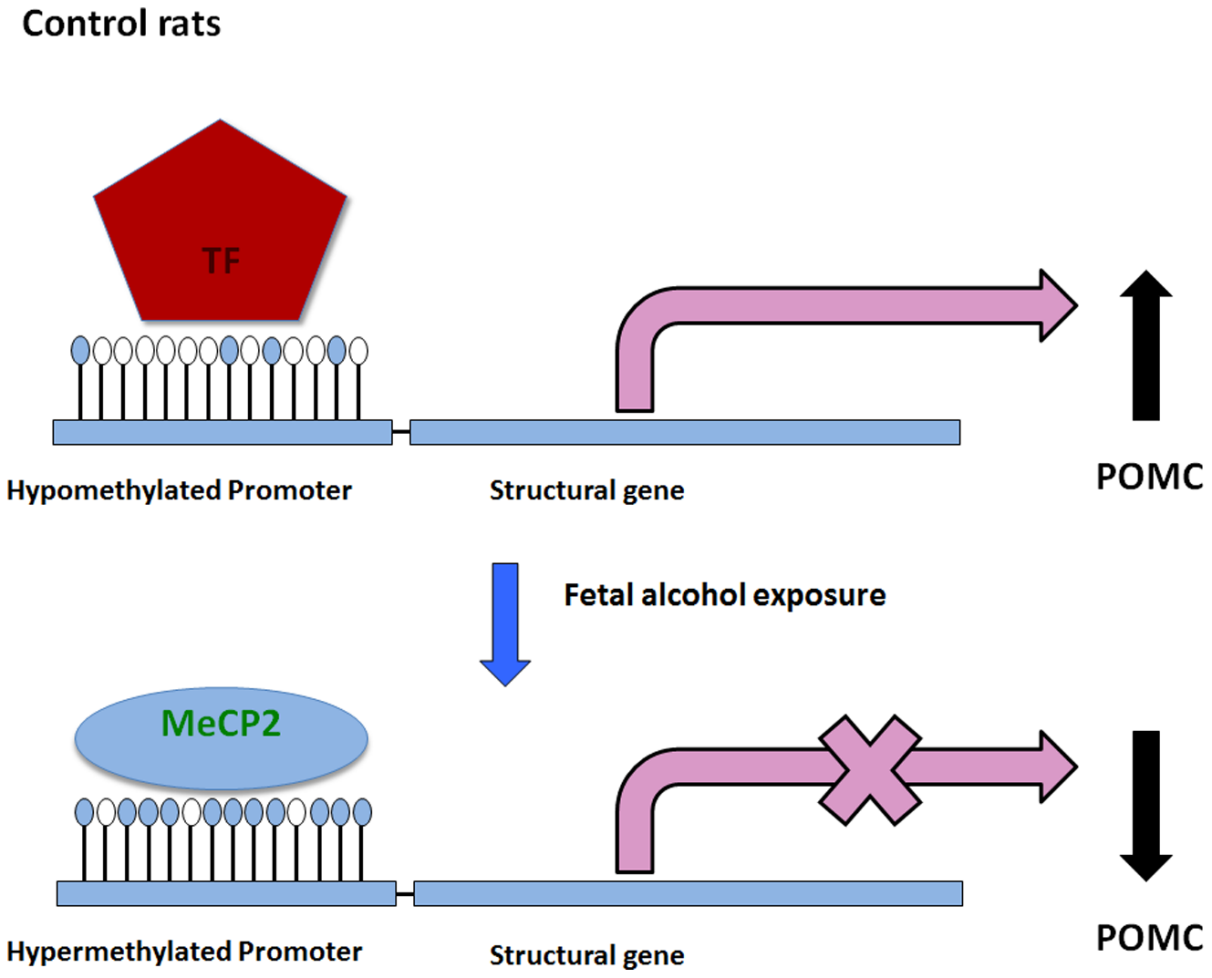
A Model illustrating the role of promoter methylation and MeCP2 binding in regulation of POMC expression following fetal alcohol exposure. In control rat offspring transcription factor binds to hypomethylated POMC promoter and activates transcription (top). Fetal alcohol exposure promotes hypermethylation of the POMC promoter and recruits MeCP2. MeCP2 binding to CpG methylated POMC promoter prevents the transcription factor's ability to bindto activate gene transcription.

DNA methylation at CpG dinucleotides of gene promoters is a powerful means of gene silencing [35]. Fetal alcohol exposure induces aberrant changes in DNA methylation patterns with associated changes in expression of developmental and imprinting genes [36]. We recently reported fetal alcohol exposure increased POMC promoter methylation to reduce its transcript expression [21]. MeCP2, a member of the methyl CpG binding domain (MBD) containing family of proteins, binds methylated DNA and represses transcription by recruiting histone deacetylases (HDACs) [26], [37], [38] and histone methyl transferase (HMTs) [39], [40]. MeCP2 also targets several neuronal genes including BDNF and IGFBP3 [41]–[43]. Methylated CpG dinucleotides with adjacent A/T-rich sequences are putative MeCP2 binding sites [27]. The proximal POMC promoter contains a number of A/T-rich stretches in the vicinity of CpG sites. Loss of MeCP2 expression results in a loss of interaction of MeCP2 with methylated CpG sites at the promoter, thereby upregulating the expression of a subset of genes in Rett syndrome in mouse models as well as in human patients [44], [45]. In our study lentiviral knockdown of MeCP2 expression in hypothalamic neurons results in the normalization of fetal alcohol exposure induced POMC gene silencing. However, MeCP2 knockdown did not alter POMC expression in hypothalamic neurons of controls although MeCP2 shRNA efficiently reduced its expression. The reason why MeCP2 knock down did not change POMC expression in AD, PF rat offsprings been it is known to recruit on to hypermethylated promoter to repress the transcription. The unmethylated or hypomethylated CpG islands were found to be devoid of MBDs as it has been reported for some tumor suppressor genes [46]. These results support the hypothesis that fetal alcohol exposure increases MeCP2 binding to CpG methylated POMC promoter and thereby prevents the transcription factor's ability to bind and activate gene transcription (Fig. 6). How MeCP2 recruits HDACs or HMTs onto the methylated POMC promoter to repress transcription in the fetal alcohol exposed condition is not known and it needs to be further investigated.
